# Adaptation and Validation of the MapMe Body Image Scales in Spanish Parents of Schoolchildren

**DOI:** 10.3390/children11040448

**Published:** 2024-04-08

**Authors:** Patricia Inclan-Lopez, Maria Martinez-Andres, Angela R. Jones, Martin J. Tovée, Ashley J. Adamson, Raquel Bartolome-Gutierrez

**Affiliations:** 1 Social and Health Care Research Center, University of Castilla-La Mancha, 16071 Cuenca, Spain; patricia.inclan@alu.uclm.es; 2 Faculty of Nursing, Universidad de Castilla-La Mancha, 02071 Albacete, Spain; raquel.bartolome@uclm.es; 3 Population Health Sciences Institute, Newcastle University, Framlington Place, Newcastle upon Tyne NE2 4HH, UK; angela.jones@newcastle.ac.uk (A.R.J.); ashley.adamson@newcastle.ac.uk (A.J.A.); 4 Human Nutrition and Exercise Research Centre, Newcastle University, Framlington Place, Newcastle upon Tyne NE2 4HH, UK; 5 Health and Life Sciences, Northumbria University, Newcastle upon Tyne NE1 8ST, UK; martin.j.tovee@northumbria.ac.uk

**Keywords:** family, children, body image, weight perception, visual scale, obesity, validation

## Abstract

Childhood overweight and obesity is a worldwide problem and to treat it parents’ detection has to be improved. The MapMe Body Image Scales (BIS) are a visual tool developed to improve parental perception of child weight in the United Kingdon (UK) based on British growth reference criteria. The aim of this study was to make a transcultural adaptation and validation of the MapMe BIS in Spain based on International Obesity Task Force (IOTF) cut offs A descriptive cross-sectional study was done. First, a translation and cultural adaptation was carried out. A total of 155 10–11-year-old children and their parents participated in this study. Children were measured to calculate their weight status, Body Mass Index (BMI), Body Fat Percentage (BFP) and Waist Circumference (WC), and their parents completed a purpose designed questionnaire about their perception and satisfaction of child’s body weight status using the adapted BIS. Test-retest reliability, criterion validity and concurrent validity of the adapted BIS were analyzed. This study shows that the adapted MapMe BIS has good psychometric properties and is a suitable visual scale to assess parental perception of weight status in 10 and 11-year-old children in Spain.

## 1. Introduction

Overweight/obesity in childhood is a public health problem worldwide. Children with overweight/obesity are found to have higher rates of disability, noncommunicable diseases and deaths than underweight children [1,2,3,4]. The worldwide prevalence of overweight and obesity among children aged 5–19 years rose from 4% in 1975 to just over 18% in 2016 [5]. In addition, in 2020 39 million children under the age of 5 years were identified as having overweight/obesity across the world [5]. The ALADINO study reported a prevalence of 26.2% for overweight and 18.3% for obesity in children aged 6–9 years in Spain in 2011 [6]. This study was repeated in 2015 and 2019, with a slightly lower prevalence of overweight and a similar prevalence of obesity, at 23.3% and 17.3% respectively, in 2019 [6]. Interventions are required to mitigate overweight and obesity in this demographic group, with an estimated 77% of children with overweight becoming adults with obesity, a weight status associated with a higher risk of premature death and disability [5,7,8,9,10].

Childhood obesity is preventable. Parental involvement is essential in addressing the issue. Parents play a key role in the development and maintenance of their child’s health related behaviours. Parents are also relied upon to recognise overweight/obesity in their child and take the appropriate action and/or seek support for normal weight maintenance [1,3,5,10,11,12]. Specifically, if parents do not perceive that their child is overweight, behavioural changes to modify the child’s weight development will not occur [4]. This fact highlights the importance of parental perception of child’s weight [2,4,10,12]. Mareno et al. [10] have provided a definition of parental perception of child weight: “a parent’s judgement of their child’s body weight formulated by a parent’s recognition of body size, physical appearance, functional abilities, psychosocial effects and health effects related to current body weight”. However, evidence suggests that parents’ recognition of children with overweight/obesity is limited [1,10,12,13,14,15,16].

Parents often misinterpret the weight status of their children. Recent reviews suggest that, despite the existence of societies with very different ideas of healthy weight, underestimation of child weight status is a common phenomenon amongst parents of children with overweight and obesity globally. A great variability has been observed regarding the misperception of child weight status, ranging from 12 to 71% all over the world [13,17,18,19,20,21,22], including 71% in Spain [22]. Some studies have demonstrated a correlation between the parental misperception of their child’s weight status and less healthy diets. Paradoxically, children who are perceived as above parental ideal tend to gain more weight than children whose parents think they are about the right weight. This may be due to failed weight loss attempts leading to future weight gain [15].

One possible explanation of misperception is that due to the increase in levels of overweight/obesity in childhood, there has been a shift in what is perceived as a ‘normal’ weight towards higher weight categories, which makes it difficult to identity overweight and obesity and prevents parents from taking appropriate action [10,13]. The theory of idealization could be another explanation for parental inaccuracies, which claims that people perceive attributions in a manner which is more favourable than accurate. Thus, if the body size of the children is idealised to be ‘normal’ by parents, they could have difficulties in recognising discrepant sizes [23]. This might lead to a misperception of overweight or underweight bodies as being of ‘normal’ weight [15,23].

According to Cash et al. [24], body image is a complex psychological experience of embodiment that is influenced by a variety of factors, including an individual’s beliefs, thoughts, behaviours and feelings [14,24]. Body image could be measured through the difference between the perception of current and ideal body size, indicating a degree of dissatisfaction [14]. Applying these concepts to parental satisfaction of children weight, parental satisfaction of child weight depends on parental perception [14,23,25,26,27]. Therefore, if a child with overweight is perceived as having a ‘normal’ weight, it is more likely that the parent will be satisfied [23]. The main issue with parental misperception is that parents are satisfied with their child’s body size when it is perceived as ‘normal’ or like their ideal, regardless of their actual weight status. This could lead to a lack of changes in health-related behaviours [23]. Conversely, if parents are unsatisfied, it could lead to inappropriate feeding behaviour and encourage a healthy weight child to lose or gain weight, or eat less or more [15,17,23].

Furthermore, attitudes to body image in the family are significant because parents are an important influence on children, perhaps more so than the media or peers, which became more influence in puberty [15,25,26,28,29,30]. These behaviours and excessive messages from the parents concerning body image may lead to children internalising their parents’ weight ideals. Thus, a child could idealize a lower weight if their weight is higher than his/her parents’ ideal (irrespective of his/her actual weight status) which may contribute to the development of body shape and weight concerns, and be a trigger for future eating disorder problems [15,25,26,28,29,30]. This shows the importance of the role of parents and the need for a better understanding of parental perception and satisfaction of children’s weight. Additionally, when studying body image and comparing different studies there is a problem in the lack of consistency in measurement tools and the limited range of validated measures. Moreover, it is important to provide a tool that facilitates the accurate perception of children’s weight, because without recognition it is unlikely that parents will modify the children’s lifestyle or commit to behavioural interventions [13,31,32]. Parental satisfaction with the child’s weight is related to the internalisation of weight ideals in children and their involvement in eating disorders [15,25,26,28,29,30], hence the reason why there is a need for a tool to assess this issue.

The literature suggests that non-growth-chart-based approaches should be considered, and given the fact that parents tend to determine child weight status via visual assessments [8,33], the use of a visual tool could improve the assessing parent perception and satisfaction. Also, a visual tool may have potential for interventions aimed at improving parental recognition of childhood overweight and obesity. A fast and reliable tool with images that correspond to a specific Body Mass Index (BMI) is required, so that parents can make a visual comparison and thus avoid subjective evaluations through words. Moreover, there are no visual scales created using the IOTF cut points [34], a measure used worldwide to identify child weight status, so that it could be used in different countries. There are some visual scales created about schoolchildren’s self-perception of body image, but there are no parental visual scales about children weight, to the best of our knowledge. The MapMe Body Image Scales (BIS), created in the United Kingdom (UK), was chosen, and presents visual images of children aged 4–5 and 10–11 years ranging from underweight to very overweight. They are the first sex- and age-specific BIS of children, based on British growth reference (UK90) criteria [31]. These scales were designed as part of the MapMe Tool used in the MapMe study of parent perceptions and child weight outcomes. MapMe is an intervention that includes both the BIS and supporting information on healthy eating, physical activity and sources of support. We considered that the above scales could be useful in assessing parents’ perception of their children’s weight in general, not only in relation to the intervention for which they were designed [16].

In Spain, parents’ perception of their child’s weight status has been the subject of research through the Spanish National Health Surveys [22]. Parents provide their child’s weight and height and are asked to judge their weight status. A potential weakness in this approach is that there is no way to verify the accuracy of the values given for the child’s height and weight, and therefore the accuracy of the weight categorisation. Parental satisfaction of child weight has not been the subject of research in Spain, to the best of our knowledge. There is no visual scale that measures the perception and satisfaction of the weight of children in Spain, one approach requires a cross-cultural adaptation of the MapMe BIS from the UK [33,35,36,37]. Considering the above, the main objective of this study was to adapt and validate the BIS from the MapMe Tool, using the IOTF cut points, for children aged 10–11 years old in Spain. The secondary objective was to know parental perception and satisfaction of children’s weight. In addition, the questions that the authors of MapMe Tool used to assess its impact were added. This study set out to test the following hypotheses:

- The correlation between the parental perception of their child’s weight status using the adapted MapMe BIS and the objective measures of child weight status (BMI, Body Fat Percentage (BFP) and Waist Circumference (WC)) will be high.
- This correlation will be greater than between the other perception questions (verbal and analogical) and these weight status variables.
- Parental perception of their child’s weight status using the adapted BIS will be more strongly correlated with the objective measures of child weight status than parental satisfaction with their child’s weight status.

## 2. Materials and Methods

### 2.1. Study Design

Transcultural adaptation and validation of the MapMe BIS were conducted through a cross-sectional study.

### 2.2. Participants

This study was carried out with children aged 10 and 11 years enrolled in schools in the city of Albacete, Spain, and their parents. Children aged 10–11 years and their parents were invited to take part: participation involved the children being measured and the parents signing the consent form and completing the MapMe BIS. The inclusion criteria were as follows: 10–11 year-old children who could be measured without problems (i.e., could stand un-aided) and the completion of a signed written consent form by parents. The exclusion criteria were not having signed written consent, not being able to be measured, and ages different than 10 and 11 years.

318 children were invited to participate. A final sample of 155 children was achieved. This was due to:

- A total of 4 individuals were excluded from the study due to being outside of this age group, one for not being able to be measured due to a mobility problem.
- A total of 117 children did not provide signed parental consent.
- A total of 41 parents did not complete the questionnaire or answered only some of the questions.

A subsample of 54 participants was included in the test-retest reliability assessment. The test-retest version of the tool was completed by 49 parents, whose children were boys in 24 cases and girls in 25 cases.

### 2.3. Procedure

Six public schools in the city of Albacete, Spain, were selected through cluster sampling to recruit participants. To ensure socio-economic diversity, the schools were located in five different districts of the city. The participants in this study were recruited from January to April 2019. Data collection was carried out in school facilities from May to June 2019.

The Department of Education, Culture and Sport of the Council of Communities of Castile-La Mancha supported this project. They sent a letter to schools to inform them about the study. Researchers visited each school and briefed schools’ principals and tutors with regards to all of the study details and the confidentiality of the data, and permission was obtained to carry out study procedures in the selected schools. Upon the agreement of tutors to support the study, children were asked to participate by classroom-by-classroom briefings. Information sheets and consent forms were given to tutors, who were responsible for distributing those documents to families via their children’s school bag requesting parental completion. Parents were offered a meeting to resolve doubts in the information sheets. Only one parent’s group from a school reported doubts, which were resolved in a meeting. On information sheets was detailed that children had to return the consent form and the completed parental questionnaire (adapted BIS and questions described below) on the measurement day.

Another copy of the BIS and questionnaire was re-administered to parents in the same way after 10–15 days to assess the test-retest reliability.

The study protocol was approved by the Human Research Ethics Committee of the Hospital of Albacete. The participants’ right to anonymity was complied with.

### 2.4. Instruments

#### 2.4.1. Anthropometric Measures

All measurements were taken under standardised conditions, with two non-consecutive measurements completed by the same researcher.

- Weight: In kilograms, using a TANITA^®^ BC-418 MA bioimpedance scale.
- Height: In centimetres was measured using a SECA^®^ 222 wall height rod.
- Weight status: based on BMI as per the IOTF criteria.

#### 2.4.2. Gold Standard Measures for Validation

- BMI: Calculated as “weight (kg)/(height (m)^2^)”
- BFP: determined by TANITA^®^ BC-418 MA bioimpedance scale.
- WC: Measured using a flexible tape at the midpoint between the iliac crest and the last rib.

#### 2.4.3. Parental Questionnaire

The parental questionnaire was used to assess the parental perception and satisfaction with child weight. It consisted of adapted MapMe BIS and of four questions: two questions referred to the adapted MapMe BIS, and two added questions without these images. These questions are the same ones that were used to assess the impact of the MapMe intervention in UK.

One of the questions which referred to the adapted MapMe BIS was about parental satisfaction with child weight and another one about parental perception of child weight. The other two were a categorical question and a visual analogue scale, regarding parental perception of child weight. The difference between these two questions is that the categorical question is discreet, and the visual analogue scale is continuous. In addition, the categorical and visual analogue scale questions were used to verify the concurrent validity of the adapted MapMe BIS.

- Adapted MapMe BIS: it was based on the original body images of children created by Jones et al. [31] using British growth reference (UK90) criteria. These body stimuli were based an analysis of 388 3D scans of children aged 4–5 and 10–11, to produced anatomically accurate illustrations of UK child weight categories [31]. For the validation in Spain, the images were modified to illustrate the IOTF criteria, a measure used worldwide to determine child weight status [34]. For the creation of the original tool in the UK, a total of 12 qualitative sessions were held with parents and childhood obesity health professionals. The result was a sex- and age-specific BIS from two angles: front and profile. Following the format of the original BIS [31], the adapted MapMe BIS was composed of seven different bodies which correspond to seven weight categories: (A) underweight; (B) lower-healthy weight; (C) mid-healthy weight; (D) upper-healthy weight; (E) overweight, (F) lower-very overweight and (G) upper-very overweight. For the analysis, these categories were grouped into four subcategories as per the IOTF: A and B for underweight, C and D for healthy weight, E and F for overweight, and G for obesity.
- Question referred to adapted MapMe BIS: in the first question, called visual perception scale, parents had to choose which of the body shapes best represented their child; the second question, called visual satisfaction scale, analysed satisfaction or dissatisfaction, by asking the parents which image they would like their child to look like/to resemble/seem. These two questions create two variables: perception, that could be correct (if the weight category of the figure coincides with the child’s objectively measured real weight status measured by us) or incorrect (if the weight category of the figure does not coincide with the child’s objectively measured weight status); and satisfaction, that could be satisfied (if the figure chosen coincides with the figure chosen for the visual perception scale of perception), or unsatisfied (if the figure chosen does not coincide with the figure chosen for the visual perception scale if the figure is over or below the perception’s figure).
- Perception based on categorical question: This perception question, with four categorical response options, asked the following question: “How would you describe your child’s current weight at the moment?: underweight, normal weight, overweight or obese” [38].
- Perception based on visual analogue scale: The other perception question had a visual analogue scale response option, consisting of a 10 cm line with one aspect being ‘extremely underweight’, and the other aspect being ‘extremely overweight’. Parents had to specify the position along the line which best described their child’s weight.

#### 2.4.4. Transcultural Adaptation of the Questionnaire

- The inverse translation method was applied to the questions used to assess the impact of the MapMe intervention by specialised English translators. Two bilingual specialists translated the parental questionnaire into Spanish and a native English speaker with Spanish language knowledge carried out an inverse translation. The three translators compared the inverse translation with the original version to determine the degree of semantic and cultural equivalence. Content validity was assessed using the Delphi method, by a team of experts which was composed of 2 paediatricians, 3 paediatric nurses, 1 school nurse, and 2 child psychologists.
- The final version of the questionnaire (adapted BIS and additional questions) was administered to 10 mothers as a pilot comprehension test of the instrument, and no problems were reported. No one proposed any changes, all agreed with the figures of the scale (hair, eye, and skin colour) and considered that it correctly represented the BMI.

### 2.5. Statistical Analysis

The IBM SPSS Statistics programme (Version 24.0) was used for data analysis. First, the descriptive characteristics of the study sample were analysed. The significance level was set at *p* < 0.05. Descriptive statistics of the quantitative variables are presented as the mean, standard deviation, range and confidence interval at 95%. To assess test-retest reliability, the same questionnaire was administered to a subsample after ten days to analyse sensitivity by the intraclass correlation coefficient (ICC) with an ICC value above 0.8 considered good reliability and 0.9 excellent reliability [39]. To assess the criterion validity of the adapted MapMe BIS, correlations were analysed between the visual perception scale score and the three criteria that evaluated objective child weight status: the child’s BMI, BFP and WC; and with the two further perception questions analysed using ANOVA. Tukey’s HSD Test for multiple comparisons was then used to compare the mean value of these variables by sex. The relationship between the visual perception scale, the visual satisfaction scale and their association was studied. Correlation was used to determinate the relationship between parental satisfaction according to the chosen image (adapted BIS), objective weight status and objective measure. It was analysed when the answer was ‘unsatisfied’ i.e., when the parent chose a figure which did not coincide with the figure chosen for the visual perception scale.

## 3. Results

Table 1 shows the descriptive characteristics of the sample by age, sex and weight status. The sample was composed of a total of 155 children, where 87 were 10-year-old (56.1%) and 68 11-year-old. There were 77 boys (49.7%) and 78 girls (50.3%). Researchers measured the children and classified their weight status following the IOTF criteria. There was a total of 18 children classified as underweight (11.6%), 95 as healthy weight (61.3%), 32 as overweight (20.6%) and 10 as obese (6.5%). As shown in Table 2, score ranges, means and standard deviations for each variable were calculated. BMI, BFP and WC of the sample by sex are shown in Table 2, that shows that girls had higher BFP scores than boys, whereas they had lower mean WC. There were only significant differences by sex for BFP (IC.95, *p* = 0.005) and WC (IC.95, *p* = 0.044).

**Figure 1:**
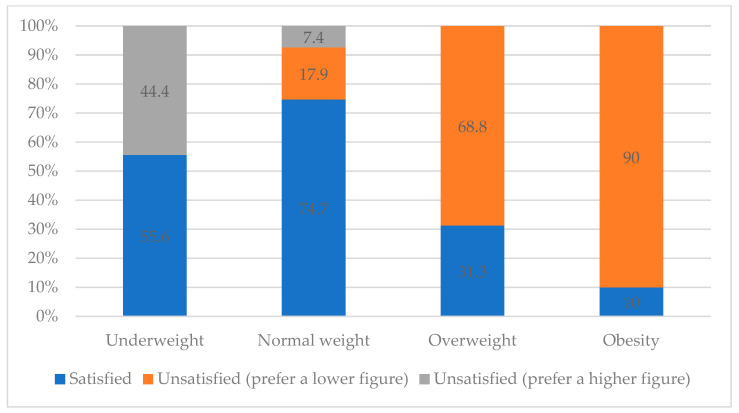
Parental satisfaction of child weight by weight status.

Table 3 shows the relationship between objective child weight status and the parents’ perception of their child’s weight status using the adapted MapMe BIS (visual perception scale). This table shows the lack of agreement between the figure chosen by parents in the perception question using the adapted MapMe BIS and the child’s weight status according to an objective measurement (BMI). Other relevant data was the parental satisfaction of the child’s weight by weight status, which is shown in Figure 1. As can be discerned in the figure, the parental satisfaction variable was classified into three options: satisfied, unsatisfied (preferring a lower figure) or unsatisfied (preferring a higher figure). This figure shows that dissatisfaction with normal weight is lower. This dissatisfaction with normal weight is shared by parents who prefer their children to be in a high or low category. Moreover, parental dissatisfaction is higher in children classified with obesity than with underweight and overweight.

### 3.1. Test-Retest Reliability

The test-retest reliability coefficient was calculated using a subsample of 49 parents who completed the questionnaire again 10 days later. A total of 54 parents were invited to participate but five (5) did not complete the questionnaire. The intra-class correlation coefficient (ICC) was 0.934.

### 3.2. Criterion Validity

A one-way ANOVA was carried out to compare the relationship between parental perception using the adapted MapMe BIS, and the three variables objectively assessing weight status: BMI (F: 49,661, sig: 0.000), BFP (F: 56.006, sig: 0.000) and WC (F: 34.981, sig: 0.000) as dependent variables, by sex. All three variables showed a statistically significant difference between at least two groups.

Tukey’s HSD Test for multiple comparisons identified that the mean value of perception was significantly different between all categories of weight status in the three variables in boys at *p* < 0.05. It is important to note that parents distinguished two categories in girls in parental perception variable using the adapted BIS: underweight and normal weight; and overweight and obesity. There were only significant differences between underweight and weight in body fat percentage, in the other categories parents distinguished the two aforementioned groups.

Differences in the mean score of the variables BMI, BFP and WC by actual weight status categories, by sex, are shown in Table 4. A higher weight status category was significantly associated with a higher mean of the three ponderal variables.

### 3.3. Concurrent Validity

Table 5 shows correlations between all questions on the parental questionnaire, objective weight status variables and actual weight status controlled by sex and age. The correlation between the visual perception question using the adapted MapMe BIS with all weight status variables was higher than the categorical l and visual analogue scale perception questions.

As shown in Table 6, the visual scale resulted in a higher percentage of parents correctly perceiving the weight of their underweight, overweight and obese children when compared to the categorical and visual analogue scale questions.

### 3.4. Satisfaction Visual Scale

The validation of the satisfaction visual scale was carried out through correlation with the perception scale, as there was no satisfaction categorical question. Satisfaction behaviour was likewise studied with gold standards and visual analogue scale perception questions. The correlation of the satisfaction questions was lower than the correlations of visual perception questions with all variables. There was no statistically significant difference in parental satisfaction by sex.

## 4. Discussion

The objective of this study was to adapt and validate the MapMe BIS for determining parental perception of child weight in Spain. It is the first time when MapMe BIS has been validated for use in other than English language. This scale has been previously designed in the UK for use in an intervention aiming to prevent childhood overweight and obesity. In this modified version, we added four questions about parental perception and satisfaction with their child’s weight. The results indicate that the scale has good psychometric properties. Regarding reliability, the test-retest ICC was greater than 0.9, which is considered to be almost perfect by some authors [39,40,41], indicating satisfactory temporal stability. In terms of criterion validity, the ANOVA showed significative differences between the different weight status categories of the modified BIS and the objective weight measures (BMI, FBP and WC). Additionally, the results showed that the adapted MapMe BIS is a more suitable method for assessing parental accuracy perception of the weight of 10–11-year-old children in the Spanish population, compared to categorical and visual analogue scale perception questions.

As outlined in the introduction, it is important to develop a visual scale for the assessment of parental classification of child weight status and to analyse parental satisfaction of child weight status in Spain. The incidence of overweight and obesity levels in childhood has increased, and parents play a crucial role in addressing this issue. One of the most challenging problems is the parents’ failure to identify childhood overweight and obesity and so take action and/or seek professional help. This study found that 36.8% of the sample misperceived their child’s weight using the adapted BIS. Children classified as obese were perceived accuracy in 60% of cases, and the rest was classified as overweight. Furthermore, 26.3% of parents tend to misclassify their children when they have a normal body weight. This fact emphasized the necessary parental education about body weight and the need to improve parental perception, even when children have a normal weight. Categorical and visual analogue scale questions are more accurate when examining responses from parents with healthy weight children. However, parents’ perception using the adapted BIS has a higher accuracy rate when examining responses from parents with underweight, overweight and obese children. This is interesting given that the main issue is the misperception of these weight statuses. While the correct perception of healthy weight is important, it may not be as crucial as the other weight categories, as children in those categories require changes in their lifestyles.

This scale has many advantages, one of which is its objectivity compared to other scales that rely solely on subjective evaluations. It is a visual scale that allows parents to classify their child based on visual comparisons rather than categorical descriptors. This can be particularly useful because parents may not understand the differences between the various weight status categories described by words but can more easily compare their children to the image on the scale. Parents’ ability to recognize overweight or obese children is limited, possibly due to the use of visual assessments and comparisons with other children, which can be influenced by personal, cultural, and social factors. Objective measures, such as BMI, should be used instead. The study results confirm that the correlation between the adapted BIS and the variables of the children’s objective weight is stronger than that of the other perception questions (categorical and visual analogue scale). This indicates that this scale is more accurate than the other perception questions with which it was compared.

Additionally, the images on the scale were created by averaging 3D scans of real children classified in each weight status category to provide an accurate visual representation. It may also be beneficial to visualise the thickness of the arms, legs and abdominal area since the scale is 3D and allows for both a front and profile view. The validation of the adapted BIS was compared to gold standard variables, and anthropometric data measurements were conducted to avoid self-reported measurement biases. In the original MapMe BIS, images were created based on the UK90 criteria [42]. In our validation, we modified the images to conform to the IOTF criteria [34]. Once validated, these images could be used in other countries.

Additionally, this parent questionnaire is simple to administer and complete, and can be used in both paper and digital formats. It could be suitable for managing and identifying overweight/obesity in primary care and monitoring children’s lifestyles. It could be included in national epidemiological surveys or completed by children to explore self-image and self-assessment of body image [31]. Another potential use could be an intervention to improve parental perception, as assessed by the original authors of the MapMe BIS [16]. A study was conducted to verify the effectiveness of an intervention using the MapMe BIS to improve parental perception of overweight and very overweight children. The study aimed to reduce age-relevant BMI z-score 12 months post-intervention. It was found that the BMI z-score decreased at 12 months, but there was no direct evidence of improvement in the parents’ ability to correctly categorise the child’s overweight status. Future research is required to investigate interventions using the body scales and their effectiveness in supporting improved BMI z-score [16]. These results could conclude that the BIS is a useful tool for evaluating parental perception.

This study has identified other interesting issues. The ANOVA showed significant differences between the different weight status categories of the adapted BIS and BMI, BFP and WC. These differences were observed by sex. A post hoc test revealed significant differences between all categories (underweight, normal weight, overweight and obese) for all objective weight variables in boys. These findings suggest that parents could use the scale reliably for all weight statuses in boys. However, our findings suggest that the parents’ perception of girls differed. No differences were found between underweight and normal weight, and neither between overweight and obesity for this group. Interestingly, parents distinguished between the two weight categories: one for the two lowest categories and another for the two highest. These results may be due to concerns about the girls’ image or weight. Overweight is a concern for parents, not only in the case of obesity, especially with regards to boys. Certain studies have shown that parents tend to pay more attention to the weight of boys more than girls [13,43,44]. This could be due to sex differences in body composition, but there are also social norms that view boys with larger and athletic bodies as having a physical advantage, while girls may be pressured to have a smaller body size [13,43,44]. This situation may cause boys to be more likely to abstain from food if they are overweight or obese, and girls to idealize a smaller body size, putting them at greater risk of developing an eating disorder [13,43,44].

As previously mentioned, the most precise question on the parental questionnaire used in this study was the visual perception question (using the adapted BIS), followed by the categorical question and then the visual analogue scale question. Consistent with the hypothesis, the correlation between satisfaction and BMI, BFP, and WC is lower than the correlation between perception and BMI, BFP, and WC. This is because satisfaction does not measure the perception of the child’s weight, but rather represents an ideal. Since satisfaction is an ideal construct and has been compared to objective measures, it is important to note that anything based on an ideal may differ from a more biometrically accurate representation. The hypothesis was that parents whose children’s weight was classified as obese would have lower satisfaction than those with other weight statuses. Our observations indicate a negative correlation between satisfaction and child weight classification. Parents of healthy weight children reported higher satisfaction levels, with 74.7% expressing satisfaction, compared to only 10% of parents of obese children. Parents who classified their children’s weight as healthy, regardless of the accuracy of their perception, were more satisfied than parents of underweight, overweight, or obese children who accurately or inaccurately classified their children’s weight.

Limitations include the fact that the study was carried out in schools from Albacete. However, this population is typical of the school population in the rest of Spain, which allows for the results to be extrapolated more widely. Additionally, as this is the first time the scale has been adapted and validated in another culture, these results cannot be compared with a previous validation. Another limitation is the research design, which might have been better suited to a longitudinal study to analyse predictive validity. The sample size is relatively small, and this validation is only available for 10 and 11-year-old children. These ages were chosen because obesity after puberty persists in adults in 40–70% [45], and it was considered that in an intervention, this age group would be better able to adopt healthy habits than younger children. Therefore, helping parents to recognise weight problems would be more likely to lead to improved child weight status. However, further research is required to create and validate figures for other age groups.

It is important to mention the non-response rate, approximately 50%, in these surveys. Specifically, there were more individuals who did not answer the satisfaction question compared to the perception question. This could be due to the controversial and perhaps fraught nature of parental satisfaction regarding their child’s weight. Another possible explanation could be that parents did not understand the difference between the perception and the satisfaction question. However, a pilot comprehension test and Delphi method of the instrument were conducted, and no issues were reported. Additionally, the final sample is within the usual size range for validation studies [46] and it is considered sufficient for this specific study given that the scale has only 4 items and no factor analysis is required.

## 5. Conclusions

In conclusion, the adapted BIS is a useful visual scale for identifying parental misperception of weight status in 10- and 11-year-old children in Spain and it seems to function. This study has validated an adapted version of the BIS, which has good psychometric properties. This scale would be widely for use in public and clinical health practice, as well as being an aid for future scales. This validation could be useful for future research studies comparing children from Spain and the UK.

## Figures and Tables

**Table 1:** Descriptive characteristics of the sample by age, sex and weight status.

		*n*	%
Age (years)	10	87	56.1
	11	68	43.9
Sex	Male	77	49.7
	Female	78	50.3
Weight status	Underweight	18	11.6
	Healthy weight	95	61.3
	Overweight	32	20.6
	Obese	10	6.5
Total		155	

**Table 2:** Body mass index, body fat percentage and waist circumference of the sample by sex.

		Male		Female		Total	Sig
Age (years)	10	46		41		87	0.371
	11	31		37		68	
Weight status							0.528
	Underweight	12		6		18	
	Healthy weight	45		50		95	
	Overweight	14		18		32	
	Obese	6		4		10	
		Mean	SD	Mean	SD		
BMI		18.91	3.68	18.73	3.27	0.750	
Body fat percentage		19.33	7.41	22.78	7.65	0.005 *	
Waist circumference		67.75	8.75	65.06	7.69	0.044 *	

Body mass index (BMI); body fat percentage (BFP); waist circumference (WC), Standard deviation (SD). CI 0.95, sig *p* < 0.05 *.

**Table 3:** Relationship between actual weight status and category of parental weight perception (adapted BIS) *n* (%).

Visual Parents Perception of Their Children Body Weight	Objective Weight Status				
	Underweight	Normal Weight	Overweight	Obese	Total
Underweight	12	13	0	0	25
	66.7	13.7	0	0	16.1
Normal weight	5	57	3	0	65
	27.8	60.0	9.4	0	41.9
Overweight	1	25	23	4	53
	5.6	26.3	71.9	40	34.2
Obese	0	0	6	6	12
	0	0	18.8	60	7.7
Total	18	95	32	10	155
	100	100	100	100	100

χ^2^ = 124.625, sig 0.000.

**Table 4:** Mean score of the variables BMI, BFP and WC by actual weight status categories, by sex.

	Male			Female		
	BMI	BFP	WC	BMI	BFP	WC
Underweight	14.37	10.96	59.51	14.26	10.57	56.46
Healthy weight	17.89	17.49	65.09	17.38	20.24	61.87
Overweight	22.77	26.37	76.62	22.28	30.71	73.91
Obese	26.66	33.40	83.42	26.34	37.21	77.92

*p* = 0.000 for all variables (BMI, BFP, and WC) by sex.

**Table 5:** Correlations between categorical, visual analogue scale and visual questions of parental perception, parental satisfaction question, objective weight status variables and actual weight status controlled by sex and age.

	Categorical Question	Visual Analogue Scale Question	Visual Question	Satisfaction Question	BMI	BFP	WC	Weight Status
Categorical question	1.000	0.561	0.673	0.256	0.724	0.676	0.682	0.705
Visual analogue scale question	0.561	1.000	0.461	0.229	0.431	0.393	0.429	0.398
Visual question	0.673	0.461	1.000	0.582	0.776	0.792	0.747	0.717
Satisfaction question	0.256	0.229	0.582	1.000	0.417	0.434	0.423	0.407
BMI	0.724	0.431	0.776	0.417	1.000	0.921	0.909	0.903
BFP	0.676	0.393	0.792	0.434	0.921	1.000	0.870	0.827
WC	0.682	0.429	0.747	0.423	0.909	0.870	1.000	0.784
Weight status	0.705	0.398	0.717	0.407	0.903	0.827	0.784	1.000

*p* < 0.05 all variables.

**Table 6:** Percentage of correct parental perception of child weight status by objectively measured child weight status and question type. *n* (%).

% Correct Perception	Underweight	Healthy Weight	Overweight	Obese
Categorical question	50.0	90.5	59.4	10
Visual analogue scale question	33.3	92.6	28.1	10
Visual perception question (adapted MapMe BIS)	66.7	60	71.9	60

## Data Availability

The data presented in this study are available on request from the corresponding author. The data are not publicly available due to privacy.

## References

[1] Canals-Sans J., Blanco-Gómez A., Luque V., Ferré N., Ferrando P.J., Gispert-Llauradó M., Escribano J., Closa-Monasterolo R. (2016). Validation of the Child Feeding Questionnaire in Spanish Parents of Schoolchildren. J. Nutr. Educ. Behav..

[2] Garrido-Miguel M., Oliveira A., Cavero-Redondo I., Álvarez-Bueno C., Pozuelo-Carrascosa D., Soriano-Cano A., Martínez-Vizcaíno V. (2019). Prevalence of Overweight and Obesity among European Preschool Children: A Systematic Review and Meta-Regression by Food Group Consumption. Nutrients.

[3] World Health Organization (2016). Report of the Commision on: Ending Childhood Obesity.

[4] Ames H., Mosdøl A., Blaasvær N., Nøkleby H., Berg R.C., Langøien L.J. (2020). Communication of children’s weight status: What is effective and what are the children’s and parents’ experiences and preferences? A mixed methods systematic review. BMC Public Health.

[5] World Health Organization Obesity and Overweight: Key Facts. https://www.who.int/news-room/fact-sheets/detail/obesity-and-overweight.

[6] AESAN (2020). Informe Breve Sobre la Alimentación, Desarrollo Infantil y Obesidad en España (ALADINO 2019).

[7] Fan H., Zhang X. (2021). Recent trends in overweight and obesity in adolescents aged 12 to 15 years across 21 countries. Pediatr. Obes..

[8] Almoosawi S., Jones A.R., Parkinson K.N., Pearce M.S., Collins H., Adamson A.J. (2016). Parental Perception of Weight Status: Influence on Children’s Diet in the Gateshead Millennium Study. PLoS ONE.

[9] Ortiz-Pinto M.A., Ortiz-Marrón H., Rodríguez-Rodríguez A., Casado-Sánchez L., Cuadrado-Gamarra J.I., Galán I. (2020). Parental perception of child health status and quality of life associated with overweight and obesity in early childhood. Qual. Life Res..

[10] Mareno N. (2014). Parental perception of child weight: A concept analysis. J. Adv. Nurs..

[11] Garrido-Miguel M., Cavero-Redondo I., Álvarez-Bueno C., Rodríguez-Artalejo F., Moreno L.A., Ruiz J.R., Ahrens W., Martínez-Vizcaíno V. (2019). Prevalence and Trends of Overweight and Obesity in European Children from 1999 to 2016: A Systematic Review and Meta-analysis. JAMA Pediatr..

[12] Parkinson K.N., Jones A.R., Tovee M.J., Ells L.J., Pearce M.S., Araujo-Soares V., Adamson A.J. (2015). A cluster randomised trial testing an intervention to improve parents’ recognition of their child’s weight status: Study protocol. BMC Public Health.

[13] Warkentin S., Mais L.A., Latorre M.D.R., Carnell S., Taddei J.A.A. (2018). Factors associated with parental underestimation of child’s weight status. J. Pediatr..

[14] Warkentin S., Henriques A., Oliveira A. (2021). Parents’ perceptions and dissatisfaction with child silhouette: Associated factors among 7-year-old children of the Generation XXI birth cohort. Eat. Weight. Disord..

[15] Hahn S.L., Barry M.R., Weeks H.M., Miller A.L., Lumeng J.C., Sonneville K.R. (2021). Parental perceptions of actual and ideal body weight in early childhood prospectively predict adolescent perceptions of actual and ideal body weight among a low-income population. Eat. Weight. Disord..

[16] Jones A.R., Mann K.D., Cutler L.R., Pearce M.S., Tovée M.J., Ells L.J., Araujo-Soares V., Arnott B., Harris J.M., Adamson A.J. (2023). A Randomised Control Trial Investigating the Efficacy of the MapMe Intervention on Parental Ability to Correctly Categorise Overweight in Their Child and the Impact on Child BMI Z-Score Change at 1 Year. Children.

[17] Meredith-jones K., Williams S., Taylor R. (2016). Agreement between parental perception of child weight status and actual weight status is similar across different ethnic groups in New Zealand. J. Prim. Health Care.

[18] Kelleher E., Millar S., Shiely F., Perry I., Harrington J. (2018). RF34 Parent and child misperception of child weight status: A cross-sectional analysis of the cork children’s lifestyle study (CCLaS). J. Epidemiol. Community Health.

[19] AlHasan D.M., Breneman C.B., Lynes C.L., Callahan-Myrick K. (2018). Factors that Influence Parental Misperception of Their Child’s Actual Weight Status in South Carolina. Matern. Child Health J..

[20] Rodrigues D., Machado-Rodrigues A.M., Padez C. (2020). Parental misperception of their child’s weight status and how weight underestimation is associated with childhood obesity. Am. J. Hum. Biol..

[21] Hong S.A., Peltzer K., Jalayondeja C. (2019). Parental misperception of child’s weight and related factors within family norms. Eat. Weight. Disord..

[22] Ramiro-González M.D., Sanz-Barbero B., Royo-Bordonada M.Á. (2017). Childhood Excess Weight in Spain From 2006 to 2012. Determinants and Parental Misperception. Rev. Española Cardiol..

[23] Hager E.R., Candelaria M., Latta L.W., Hurley K.M., Wang Y., Caulfield L.E., Black M.M. (2012). Maternal Perceptions of Toddler Body Size. Arch. Pediatr. Adolesc. Med..

[24] Cash T.F. (2004). Body image: Past, present, and future. Body Image.

[25] Blowers L.C., Loxton N.J., Grady-Flesser M., Occhipinti S., Dawe S. (2003). The relationship between sociocultural pressure to be thin and body dissatisfaction in preadolescent girls. Eat. Behav..

[26] Reel J., Voelker D., Greenleaf C. (2015). Weight status and body image perceptions in adolescents: Current perspectives. Adolesc. Health Med. Ther..

[27] Rodgers R., Chabrol H. (2009). Parental attitudes, body image disturbance and disordered eating amongst adolescents and young adults: A review. Eur. Eat. Disord. Rev..

[28] Helfert S., Warschburger P. (2011). A prospective study on the impact of peer and parental pressure on body dissatisfaction in adolescent girls and boys. Body Image.

[29] Schur E.A., Sanders M., Steiner H. (2000). Body dissatisfaction and dieting in young children. Int. J. Eat. Disord..

[30] Neves C.M., Cipriani F.M., Meireles J.F.F., Da Rocha Morgado F.F., Ferreira M.E.C. (2017). Body image in childhood: An integrative literature review. Rev. Paul. Pediatr..

[31] Jones A.R., Tovée M.J., Cutler L.R., Parkinson K.N., Ells L.J., Araujo-Soares V., Pearce M.S., Mann K.D., Scott D., Harris J.M. (2017). Development of the MapMe intervention body image scales of known weight status for 4–5 and 10–11 year old children. J. Public Health.

[32] Pallan M.J., Hiam L.C., Duda J.L., Adab P. (2011). Body image, body dissatisfaction and weight status in south asian children: A cross-sectional study. BMC Public Health.

[33] Salcedo V., Gutiérrez-Fisac J.L., Guallar-Castillón P., Rodríguez-Artalejo F. (2010). Trends in overweight and misperceived overweight in Spain from 1987 to 2007. Int. J. Obes..

[34] Cole T., Lobstein T. (2012). Extended international (IOTF) body mass index cut-offs for thinness, overweight and obesity. Pediatr. Obes..

[35] Ministerio de Sanidad Consumo y Bienestar Social G. (2018). Encuesta Nacional de Salud. España 2017.

[36] Agencia Española de Seguridad Alimentaria y Nutrición (2020). Estudio ALADINO 2019: Surveillance Study on Nutrition, Physical Activity, Child Development and Obesity. https://www.aesan.gob.es/AECOSAN/docs/documentos/nutricion/observatorio/Brief_report_ALADINO_2019_NAOS.pdf.

[37] Rodríguez-Martin A., Novalbos-Ruiz J.P., Villagran-Perez S., Martínez-Nieto J.M., Lechuga-Campoy J.L. (2012). Parents perception of childhood overweight and obesity and eating behaviors, physical activity and sedentary lifestyle of their children. Rev. Esp. Salud Publica.

[38] Carnell S., Edwards C., Croker H., Boniface D., Wardle J. (2005). Parental perceptions of overweight in 3–5 y olds. Int. J. Obes..

[39] Koo T.K., Li M.Y. (2016). A Guideline of Selecting and Reporting Intraclass Correlation Coefficients for Reliability Research. J. Chiropr. Med..

[40] Landis J.R., Koch G.G. (1977). Landis amd Koch1977—Agreement of categorical data. Biometrics.

[41] Terwee C.B., Bot S.D.M., de Boer M.R., van der Windt D.A.W.M., Knol D.L., Dekker J., Bouter L.M., de Vet H.C.W. (2007). Quality criteria were proposed for measurement properties of health status questionnaires. J. Clin. Epidemiol..

[42] Wright C.M., Booth I.W., Buckler J.M.H., Cameron N., Cole T.J., Healy M.J.R., Hulse J.A., Preece M.A., Reilly J.J., Williams A.F. (2002). Growth reference charts for use in the United Kingdom. Arch. Dis. Child..

[43] Manios Y., Kondaki K., Kourlaba G., Vasilopoulou E., Grammatikaki E. (2009). Maternal perceptions of their child’s weight status: The GENESIS study. Public Health Nutr..

[44] Towns N., D’Auria J. (2009). Parental Perceptions of Their Child’s Overweight: An Integrative Review of the Literature. J. Pediatr. Nurs..

[45] Berenson G.S. (2012). Health Consequences of Obesity. Pediatr. Blood Cancer.

[46] White M. (2022). Sample size in quantitative instrument validation studies: A systematic review of articles published in Scopus, 2021. Heliyon.

